# Air Phthalate Emitted from Flooring Building Material by the Micro-Chamber Method: Two-Stage Emission Evaluation and Comparison

**DOI:** 10.3390/toxics9090216

**Published:** 2021-09-09

**Authors:** Wu-Ting Lin, Chung-Yu Chen, Ching-Chang Lee, Cheng-Chen Chen, Shih-Chi Lo

**Affiliations:** 1 Department of Environmental and Occupational Health, College of Medicine, National Cheng Kung University, No. 1, University Rd., Tainan City 70101, Taiwan; wtlin@abri.gov.tw; 2 Architecture and Building Research Institute, Ministry of the Interior, 13F., No. 200, Sec. 3, Beisin Rd., Sindian District, New Taipei City 23143, Taiwan; losc@abri.gov.tw; 3 Department of Occupational Safety and Health, Chang Jung Christian University, No. 1, Changda Rd., Gueiren District, Tainan City 71101, Taiwan; cyuchen@mail.cjcu.edu.tw; 4 Department of Architecture, National Taipei University of Technology, No. 1, Sec. 3, Zhongxiao E. Rd., Taipei 10608, Taiwan

**Keywords:** phthalate, semi-volatile organic compounds, micro-chamber method, flooring building materials, two-stage emission

## Abstract

The phthalate and semi-volatile organic compounds (SVOCs) are modern chemical substances and extensively existing in the indoor environment. The European Commission stipulated the “European Unified Test Criteria”, since 2011, for the declared specifications of building products (CEN/TS 16516), based on the “lowest concentrations of interest (LCI)”, the index pollutants, test method, and emission standard of “phthalate” and “SVOC” were specified in detail. The purpose of this study is to use six common indoor floor construction products in Taiwan (regenerated pseudoplastic rubber flooring, healthy pseudoplastic imitation wood floor, regenerated pseudoplastic rubber flooring, PVC floor tile/floor, plastic click floor, composite floor covered with carpet) to detect the changes in the concentration of phthalate emitted to the air. The ISO 16000-25 Indoor air—Part 25: Determination of the emission of semi-volatile organic compounds by building products—micro-chamber method is used to build a DS-BMEMC (glass micro-chamber: volume 630 mL), the SVOC, including phthalate, is collected in two stages, in the stable conditions of temperature 25 °C, relative humidity 50% and air change rate 2 times/h, the Stage 1 emission detection experiment (24 h) is performed, and then the Stage 2 heating-up desorption emission detection experiment (40 min air sampling) is performed, the temperature rises to 200–220 °C, the phthalate and SVOC adsorbed on the glass micro-chamber is desorbed at a high temperature to catch the air substances, the air is caught by Tenax^®^—TA and Florisil^®^ adsorption tube, and then the GC/MS and LC/MSMS analysis methods are used for qualitative and emission concentration analyses of SVOC of two-stage emission, respectively. The findings show that the floor construction materials emit nine phthalate SVOCs: DEHP, DINP, DNOP, DIDP, BBP, DBP, DIBP, DEP, and DMP, the two-stage emission concentrations are different, Stage 1 (normal temperature) emission concentration of six floor construction materials is 0.01–1.2% of Stage 2 (high temperature) emission concentration, meaning the phthalate SVOC of floor construction materials is unlikely to be volatilized or emitted at normal temperature. An interesting finding is that only S3 was detected DINP 72.6 (μg/m^3^) in stage 1. Others were detected DINP in stage 2. This might be because S3 has carpet on the surface. This implies that floor material with carpet may have an emission of DINP at normal temperature. The result of this study refers to the limited value evaluation of EU structural material standard emission TSVOC ≤ 0.1 ug/m^3^, the floor building material emissions are much higher than the evaluation criteria, increasing the health risk of users. The detection method and baseline can be used as the standard for controlling the emission of phthalate SVOC of Taiwan’s green building material labeling system in the future.

## 1. Introduction

In recent years, global warming, climate change, and extreme climate are aggravated, the consequent environmental change and health hazards have been extensively discussed at internationally important conferences and research organizations on sustainable building, indoor air, and healthy building, and the integrated building health safety issues have been brought into multinational policy making and international standard discussion. The World Health Organization (WHO) and International Organization for Standardization (ISO) began to make the Architectural Environment Standard, and proposed the Indoor Air Standard, Indoor Air Quality Guideline (WHO indoor guidelines), Healthy Housing (WHO Guidelines for Healthy Housing), Building Material Labels (green building material label, carbon footprint label) and LCCM (Life Cycle Carbon Minus). Indoor air quality involves how to reduce the personal health hazard and risk and increase the energy consumption of building ventilation in the building environment, the source pollution control (control of indoor pollution sources) and ventilation dilution (optimal ventilation) are proposed as control measures. Related international studies prove that indoor air quality will directly or indirectly affect human health. Some common respiratory diseases, ocular discomfort, and even problems in the nervous system have been proven to be significantly correlated with indoor air quality.

Up to 2021, the development of international standards for measuring parameters determining indoor air quality has been under the remit of the technical committee ISO TC 146. The committee has published 40 standards (ISO 16000 series standard) [1,2,3,4,5,6] and a number of others are under development. The international standards concerning VOC and SVOC emissions from construction products along with the analytical methods for formaldehyde (ISO 16000-3), VOCs (ISO 16000-6) chamber testing (ISO 16000-9), SVOC (ISO 16000-25), and (ISO 16000-33) are key base documents. As we know, ISO 16000-25 is part of ISO 16000 that specifies a test method for the determination of the area-specific emission rate of semi-volatile organic compounds (SVOCs) from newly produced building products or furnishings under defined climate conditions using a micro-chamber. The method can, in principle, also be applied to aged products. This measurement method is applicable to products and materials, such as board materials, wallpapers, flooring materials, insulation materials, adhesives, paints, and their combinations. ISO 16000-33 specifies the sampling and analysis of phthalates in indoor air and describes the sampling and analysis of phthalates in house dust and in solvent-wipe samples of surfaces by means of gas chromatography/mass spectrometry.

CEN/TS 16,516 is the main, new European VOC emission testing method that defines a European Reference Room. CEN/TS 16516 expression testing result data contains the concentration of individual VOCs and SVOCs, TVOC, TSVOC, and LCI-R values. CEN/TS 16516 particularly emphasizes the determination of VOCs and SVOCs, and assesses the toxicological relevance for substances of interest, compared to ISO 16000.

The latest studies in the world aimed at the building material emission, product content, environmental concentration, and health hazard of plasticizer-SVOCs in building materials, especially the phthalate esters (PAEs) in plasticizer, which dissipate to the air environment, adhere to solid particulate matter and dust, drift to the indoor environment, and enter the body through mouth, eyes, skin and breathing, causing reproductive poison, health hazard and inducing allergic asthma. PAEs are used as phthalates in many products. High molecular-weight plasticizers, such as DEHP, DINP, di-octyl phthalate (DNOP), and di-isodecyl phthalate (DIDP), are primarily adopted as phthalates in the manufacturing of flexible polyvinylchloride (PVC) products and building materials. Low-molecular-weight phthalates (LMWPs), including dimethyl phthalate (DMP), diethyl phthalate (DEP), di-isobutyl phthalate (DIBP), benzyl butyl phthalate (BBP), and di-n-butyl phthalate (DnBP), are primarily used in personal-care products, paints, adhesives, inks, and pharmaceuticals [7,8,9,10,11]. Because PAEs are not chemically bound to products, they are constantly emitted into the environment, and therefore, are widespread in the environment through direct release, migration, evaporation, leaching, and abrasion [8,12,13,14,15,16,17,18,19].

Phthalates are easily absorbed by aerosols and settled dust in the indoor environment, as demonstrated by many studies [20,21,22]. Lucattini et al. conducted a systematic review on the occurrence of SVOCs in consumer products, indoor air, and dust. This review pointed out that phthalates were detected at high concentrations in indoor dust from Canada, Kuwait, and the USA [23]. High concentrations of phthalates were found in indoor air in Chinese hospitals. Phthalates in indoor air was also found in Paris [24], Berlin [25], and Sweden [26]. Shinohara and Uchino investigated DEHP emissions to air and its transfer to house dust from a PVC sheet. The study found DEHP transfer from a PVC sheet to dust was 15 to 139 times higher than emission to air. The emission rates from a PVC sheet to air were 3.8 to 35 μg/m^2^/h, and the transfer rate to dust was 530 μg/m^2^/h [27].

A domestic study [28] investigated 49 cases of home environments; dust on the floor and sofas in the living room was collected and the concentration of phthalate was relatively high, especially the DEHP—higher than general indoor levels, meaning the investigated domestic homes were exposed to high phthalate concentration.

Due to the hydrophobicity of phthalates, it generally condenses on dust. In Table 1, we review the literature in east Asia and show the compounds profile of phthalates in dust. DEHP and DINP are the dominant pollutants on dust in the indoor environment. This trend is similar among different countries. This implies that there are pollutant sources in indoor environment. Building material could be the one source particularly the floor building material.

In 2013, the European Commission Joint Research Centre, in Germany, the Committee for Health-related Evaluation of Building Products (Ausschuss zur gesundheitlichen Bewertung von Bauprodukten—AgBB), and other EU member countries jointly published the New Test Criteria of Building Material Emission (CEN/TS 16516) [40], which was integrated with the long-term research project “Harmonisation framework for health-based evaluation of indoor emissions from construction products in the European Union using the EU-LCI concept” as regulation for healthy emissions from building materials of European countries. Throughout CEN/TS 16516, expression testing result data contains the concentration of individual VOCs and SVOCs, TVOC, TSVOC, some other parameters, and the R-value.

After the CEN technical specification (CEN/TS 16516) was issued, multiple product standards have been revised so as to include VOC emission standards, e.g., EN 14041 standard of floor covering products [41]. These standards will not redefine the test method, but refer to CEN/TS 16516, meaning the CEN/TS 16516 will be used in the specific standards of different products—a process that began in 2014.

In Taiwan, the government in order to move towards sustainable, comfortable, and low emissions in indoor environments, the Architecture and Building Research Institute (ABRI), Ministry of Interior (MOI) established and launched the Green Building Material (GBM) Label System. The GBM system consists of four aspects, including Healthy GBM, Ecological GBM, High-performance GBM, and Recycled GBM. By 2018, 2100 Labels have been conferred covering 14,861 green products. The base global market share for phthalates is accounted for by di(2-ethylhexyl) phthalate (DEHP), where at least 95% is added to PVC used as floor coverings, wall coverings, and floor tiles to ensure its flexibility. It is noteworthy that GBM labels lack of phthalate emission from building materials. The objectives of this study are to: (1) establish the micro-Chamber test system to determine phthalate emission from floor building material; and (2) investigate the component profile of phthalate in different floor building materials.

## 2. Methods

### 2.1. Literature Analysis Method

The document, research findings, embodiment data of the test methods and technical specifications about building materials emitted plasticizer and SVOCs were collected. Aiming at the ASTM and ISO standard test methods and the methods and procedures used in domestic and foreign seminars, including the VOC species and properties obtained by current experimental analysis, which are integrated to formulate the framework for analysis. The literature review and collected experimental data were analyzed, and the existing green building material labels (Taiwan), overall research, and test results of phthalate were integrated, so as to know the properties, state, and overall trend of building material emission. The correlation was analyzed, the healthy green building material label phthalate evaluation method was developed gradually, and the baseline and control strategy was suggested.

(1) $$q_{mA}=\left\{\frac{m_{1}}{q_{v,c}t}q_{VA}\right\}+\frac{m_{2}}{A_{t}}=\frac{m_{1}+m_{2}}{A_{t}}$$

where *q_mA_* is area specific emission rate (micrograms per square meter hour), *q_vA_* is area specific air flow rate (cubic meters per square meter hour), *m_1_* is mass collected in first step (micrograms), *m_2_* is mass mass collected in second step (micrograms), *q_v,c_* is air flow rate for micro-chamber (cubic meters per hour), *A* is surface area of test specimen (square meters), *t* is duration of the first phase (hours).

### 2.2. Experimental Analysis Method

The products of green building material label (Taiwan) and the products of non-green building material label were sampled. The small size building material emission test emission performance test and validation and the phthalate GC/MS, LC/MSMS test method of environmental testing were performed according to the plasticizer evaluation items of this study. The laboratory system instruments were used for qualitative and quantitative analyses to analyze the differences, so as to provide the suggested parameter values as the healthy green building material plasticizer evaluation baseline.

### 2.3. Establishment the Micro-Chamber Method

**Figure 1:**
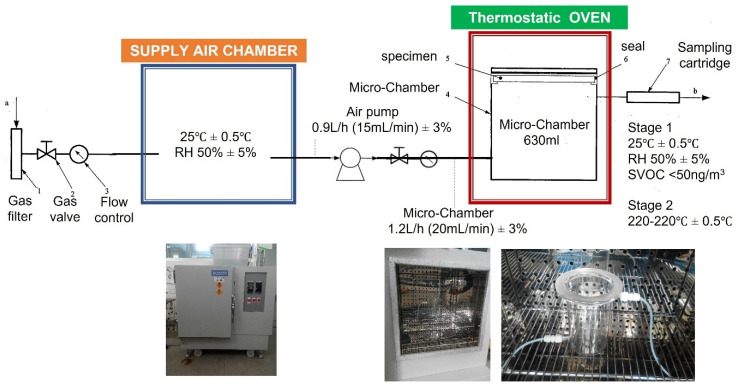
Determination of the emission of semi-volatile organic compounds by building products—Micro-chamber method.

This study builds a brand new—DS-BMEMC (determination of the emission of semi-volatile organic compounds by building products—micro-chamber method) (Figure 1). The systems and equipment are according to ISO 16000-25, and furthermore, we additional equipment such as air intake and sampling systems improved system efficiency. The DS-BMEMC system uses a micro-chamber (630 mL) through a set of testing conditions of environmental temperature, relative humidity, and ventilation (Table 2), for a 24 h sampling of SVOC and phthalate emissions from building materials and conditions. The micro-chamber (630 mL) was used for 24 h sampling of phthalate emitted from building materials in the preset ambient temperature, relative humidity, and ventilation conditions, then, the building material was taken out 24 h later, the temperature increased to 220 °C, and the SVOC and phthalate adsorbed on the wall surface are desorbed and sampled.

### 2.4. Experiment Condition

This method is applicable to the emission concentration test for SVOCs of indoor common small size building materials, including building products, the tested SVOC: an organic matter of C16-C22. The floor building material test method and experimental procedure of this study comprised two parts: (1) the simulation test was performed according to the small size environmental chamber testing method of ISO 16000-25; (2) the phthalate detection method and procedure referred to ISO 16000-33.

First, this test method was obtained by a two-stage time-dependent determination of emission test (first step test) and desorption test (second step test), which were used as qualitative and quantitative of floor building materials emitted pollutants (Table 3). During the test, in the first step, the test specimen for use in Taiwan was tested at a temperature of 25 °C and relative humidity of 50%, air change rate (2.0 h^−1^) during the emission test. The sampling time was 24 h. After completing step 1 of the emission test, the test specimen was removed, and the micro-chamber conditions increased to 220 °C for around 40 min. In this step, the sampling time should be 40 min.

### 2.5. Sampling and Analysis

Regarding the micro-chamber performance testing: the micro-chamber stability test must conform to the performance specifications of ISO 16000-25. Selection: the air vent of the micro-chamber was selected for sampling according to the studies about small size micro-chamber. Blank experiment: the blank analysis inside the micro-chamber was required before the floor building material sample test. The background concentration of the micro-chamber should be that the concentration of single SVOC does not exceed 10 μg/m^3^. Sampling planning: the phthalate emission rate of building materials attenuates with time, so the sampling time and frequency must be combined with the test sample emission characteristic as a reference frame. This study set up two stages for sampling so that the SVOCs could be thoroughly mixed in the micro-chamber. Establishment of calibration curve: 10 μL was extracted by micro airtight needle and injected into a forisil stainless steel sampling tube, desorbed by solvent, and injected into GC/MS for analysis. The ratio of peak-area of christocentrism to relative weight (ng) was made into a calibration curve. The calibration curve correlation coefficient (R^2^) must be higher than 0.995, the recovery should be within ±15%. The calibration curve range was 0.05–10 ppm according to ISO 16000-25. The GC/MS instrumental analysis method was also used, the detection limit can be5 ppb. Qualitative/quantitative analysis: after the Florisil stainless steel sampling tube was desorbed by the automatic thermal desorption device, the GC/MS was used for qualitative/quantitative analysis of the phthalates. Suggested setting conditions of instrumental analysis: ATD: referring to ISO16000-33, the selection of thermal desorption time and the setting of current carrying gas flow rate should make the alkanes desorption efficiency higher than 95%, the desorption conditions of phthalate analysis are given below: GC/MS: 60 m DB-5 MS column (0.25 mm i.d., 1.4 μm film thickness) and helium as carrier gas. The oven program was 35 °C for 1 min, ramped at 20 °C/min to 200 °C, and temperature increased from 200 °C to 260 °C at 5 °C/min, and from 200 °C to 340 °C at 20 °C/min, 4 min. A blank sample was used to monitor the contamination from the sampling and analytical process. If any blank sample was contaminated, the whole batch sample was discarded.

In this study, Florisil^®^ adsorbent tubes (produced by SKC Inc., Washington, PA, USA.) were used to sample the phthalate emission from the building materials and on the surface of micro bulkheads. Solvent desorption was by ultrasonic vibration for 30 min then through 0.22 µm membrane filtration and extraction and dissolution to GC/MS and LC/MS-MS analysis.

### 2.6. Study Floor Building Material and Target Phthalates

We chose six different flooring building materials included GB1 (wood plastic composites, WPC), GB2 (polystyrene plastic floor, PSF), GB3 (wood plastic composites, WPC), S1 (poly-vinyl chloride floor, PVC), S2 (stone plastic composite, SPC), and S3 (plastic carpet, PC) (Table 4). In Taiwan, in order to progress towards sustainable, comfortable, and low emissions in the indoor environments, the Architecture and Building Research Institute (ABRI), Ministry of Interior (MOI) established and launched the Green Building Material (GBM) Label System. The GBM system consisted of four aspects, including Healthy GBM, Ecological GBM, High-performance GBM, and Recycled GBM. In this study, the G1 to G3 flooring building materials were GBM Label, in contrast, S1 to S3 flooring building materials were non-GBM Label. Each flooring building material was cropped to 8.2 cm × 8.2 cm sample size to detect the concentration of phthalate.

The target phthalates were selected; these chemicals include DEHP, DBP, BBP, DIDP, DINP, DMP, DNOP, DEP, and DIBP.

For calculation of indoor air concentrations, the phthalate concentrations were determined from measurement solutions according to Equation (2).

(2) CA=mtubelVA

where *C_A_* is the analyte concentration in the indoor air in µg/m^3^, *m_tubel_* is the analyte mass in the thermal desorption tube in µg, *V_A_* is the sampling volume in m^3^ under sampling conditions.

## 3. Result

### Detection Result of Phthalate in Floor Building Materials

This study used the experimental analysis method (ISO 16000-25) to construct the detection technique for the phthalate emitted from green building materials. The plastic building material, three pieces of green building material label (floors-carpet, floors-regenerated plastic wood, coatings-emulsion paint), and three pieces of non-green building material label (floors-carpet, floors-PVC floor tile, coatings-emulsion paint) which may contain phthalate constituent were selected, so as to complete the small size building materials’ emitted phthalate micro-chamber test, which was compared with the plasticizer dissolution test to understand the differences and benefit of the two methods, so as to provide a reference for formulating the healthy green building material label phthalate emission detection method and evaluation baseline draft.

Table 5 shows the analytical results of phthalates in flooring building materials. All phthalates can be detected in the six building materials. Stage 2 showed higher concentrations than stage 1. The highest concentration in stage 1 was DINP (72.612 μg/m^3^) in S3. The highest concentration in stage 2 was DINP (1388.18 μg/m^3^) in GB3. The highest total concentration of phthalate was found in GB3 (1461 μg/m^3^). The lowest total concentration of phthalate was found in S2 (102 μg/m^3^). The dominant compound in these flooring building materials was DEHP, following by DINP, DBP, and BBP.

**Figure 2:**
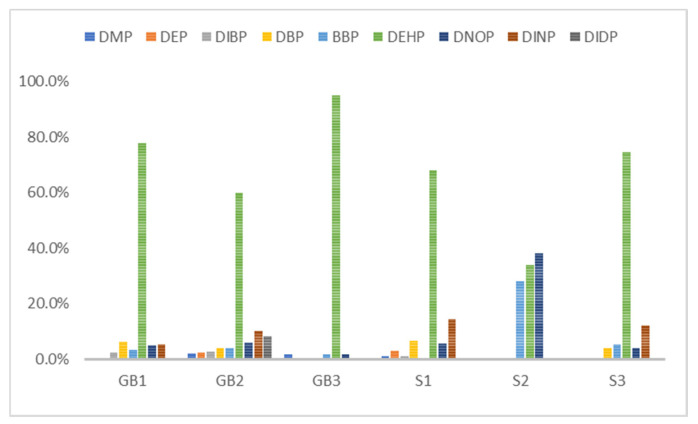
The phthalate component of six different floor building materials in this study.

For the floors building material, three pieces of Green Building Material label (regenerated pseudoplastic rubber flooring, healthy pseudoplastic imitation wood floor, regenerated pseudoplastic rubber flooring) and three pieces of non-Green Building Material label (PVC floor tile/floor, plastic click floor, composite floor covered with carpet) which may contain phthalate constituent were selected, so as to complete the small size building materials’ “micro-chamber test” to avoid the toxic SVOCs and phthalate influencing the indoor air quality. The results showed that the phthalate emission and dissolution of the experimental building products and “green building material label products” were lower than the baseline value 0.1 wt% (1000 ppm); the concentration of single phthalate emitted from the “non-green building material label products” was 34.617–442.748 μg/m^3^ (DEHP) (Table 5, Figure 2), much higher than the evaluation criteria of EU structural material standard emission TSVOC ≤ 0.1 ug/m^3^, influencing health and safety.

In Figure 2 show the target night phthalates contributed to each flooring building material, it can also be seen that DEHP in five flooring building material (GB1, GB2, GB3, S1 and S3) was very high indicating dominant phthalate source, the DEHP contributed percentage range from 33% to 95%.

## 4. Discussion

### Assessment of Validation

Phthalate substances are controlled actively in the world, and for the SVOCs with increasing health hazard, the international green building material label, the European Unified Test Criteria stipulated by European Commission, and the European new standard of building materials toxicant emission detection (CEN/TS 16516) specify the index pollutant, test method, and emission standard of plasticizer and SVOCs in detail, gradually driving the green building material label to list the plasticizer and TSVOC as necessary items of evaluation, and to set up related building materials evaluation baseline.

This study establishes the detection method for phthalate and SVOCs of building materials, referring to ISO 16000-33: standard method using GC/MS to detect phthalate, which coincides with the international building materials inspection level.

The standard novel Florisil magnesium silicate sampling analysis method established according to ISO 16000-33 can detect the phthalate and SVOCs of different flooring building materials. The detection limit of GC/MS instrumental analysis method was 100 ppb. The LC/MSMS instrumental analysis method was also used, the detection limit was 2 ppb; the Florisil magnesium silicate sampling analysis method can be used for experimental analysis and detection.

According to literature and experimental analysis, three pieces of green building material label products and non-green building material label products of boards were respectively selected from the green building material label database, including green building material label board products (regenerated pseudoplastic rubber flooring, healthy pseudoplastic imitation wood floor, regenerated pseudoplastic rubber flooring) and non-green building material label products (PVC floor tile/floor, plastic click floor, composite floor covered with carpet), to perform the emission test (nine kinds of phthalate and SVOCs emission detection). The result showed that according to the green building material generalization restricted substance phthalate content baseline 0.1% (1000 ppm), the DEHP substance emitted from regenerated green building material label products conformed to the general evaluation baseline.

However, the concentration of a single phthalate emitted from the non-green building material products (PVC floor tile/floor, plastic click floor, composite floor covered with carpet) was 34.617–442.748 μg/m^3^ (DEHP) (Table 6). According to the limited value of European structural material standard emission TSVOC ≤ 0.1 ug/m^3^, the values are much higher than the evaluation criteria, influencing health and safety.

## 5. Conclusions

The findings show that the floor construction materials emit nine phthalate SVOCs: DEHP, DINP, DNOP, DIDP, BBP, DBP, DIBP, DEP, and DMP, and the two-stage emission concentrations are different. Stage 1 (normal temperature) emission concentration of the six floor construction materials was 0.01–1.2% of Stage 2 (high temperature) emission concentration, meaning the phthalate SVOC of floor construction materials is unlikely to be volatilized or emitted at normal temperature. An interesting finding is that only in S3 was detected DINP 72.6 (μg/m^3^) in stage 1; others detected DINP in stage 2. This might because S3 has carpet on the surface. This implies that floor material with carpet might have DINP emission at a normal temperature. Furthermore, the floor building material with GBM label presented higher phthalate concentrations than products with a non-GBM label, meaning there might be a phthalate issue for the Green Building Material label in Taiwan. In overview, in Europe and America, most of the phthalates in building materials are controlled through the label system such as Green Guard and AgBB, but rarely in Asia. Currently, the control of the content of phthalates from building materials is 0.001%(*w*/*w*) 10 ppm in Taiwan EPA and 0.1% (*w*/*w*) and 1000 ppm in ABRI green building material. The results of this study imply that we can not eliminate the phthalate exposure risk from using Green Building Materials, and also can not eliminate the health risk caused by phthalate. It is suggested to fully analyze the building materials, especially floor building materials, to get full vision on this issue.

## Figures and Tables

**Table 1:** Comparison of PAEs levels (μg/g dust) in indoor dust cited in the literature by sampling locations.

AREA	Location	Sample #	DMP	DEP	DIBP	DBP	BBP	DEHP	DNOP	DINP	DIDP	Sampling Time	Studies
Southern Taiwan	Home	122	N.D.	N.D.	1.7	4.9	N.D.	298.3	81.1	77.9	32.8	2011–2014	[29]
Southern Taiwan	Elementary School	36	N.D.	N.D.	N.D.	7.9	2	860.3	212.4	436.2	43.2	2011–2014	
Southern Taiwan	Kindergarten	72	N.D.	N.D.	3.6	9.2	N.D.	571.8	180.5	346	13	2011–2014	
Southern Taiwan	Home	101	0.1	1		20.2	1	753.3				2008–2009	[30]
Southern Taiwan	Home	49	0.12	1		36.3	4.2	1505				2006–2007	[28]
Japan: Sapporo	Home	128	N.D.	N.D.	N.D.	3.1	2	1110		139		2009–2010	[31]
China: Hong Kong/Shenzhen/Guangzhou	Home	23	N.D.	N.D.	N.D.	77	4.6	1190	7.6	N.D.	N.D.	2010	[32]
China: Guangzhou/Hong Kong	Home	40	N.D.	N.D.	N.D.	13.6	0.71	773	7	N.D.	N.D.	2010	[33]
China: Nanjing	Home	215	0.1	0.2		23.7	1.6	183	0.1			2011	[34]
China: Xi’an	Home	28	N.D.		233.8	134.8		581.5				2012–2013	[35,36]
China	Home	75	0.2	0.4	17.2	20	0.2	228	0.2			2010	[37]
Korea: Seuol	Elementary School	21		N.D.		181	50	418				2005	[38]
Korea: Seuol	Kindergarten	19		N.D.		216	299	591				2005	
Korea: Seuol	Daycare center	64	N.D.	N.D.		52	50.4	3030	N.D.	946	N.D.	2012	[39]

**Table 2:** Compare ISO 16000-25 and DS-BMEMC test parameters.

Type	Item		ISO 16000-25	This Study
Test condition	temperature		23 ± 0.5 °C	25 ± 0.5 °C
	Relative air humidity		50 ± 5%	50 ± 5%
	Air flow rate	First step test	1.2 L/h	1.2 L/h
		Second step test	5.4 L/h	5.4 L/h
	Pump flow rate	First step test	0.9 L/h	0.9 L/h
		Second step test	3.6 L/h	3.6 L/h
	Specimen size		8.2 cm × 8.2 cm	8.2 cm × 8.2 cm
chamber	volume		630 mL	630 mL

**Table 3:** Two-Stage Emission method of micro-chamber (ISO 16000-25).

First Emission Stage	Second Emission Stage
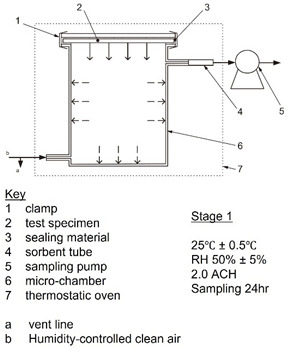	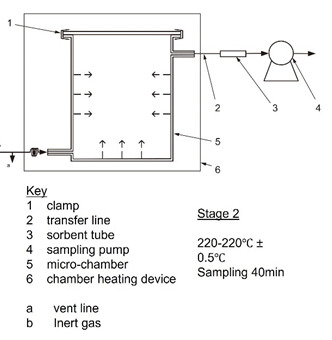
25 °C ± 0.5 °C, RH 50% ± 5%, 2.0 ACH, Sampling 24 h	220–220 °C ± 0.5 °C, Sampling 40 min

**Table 4:** Specimen of flooring building materials.

No.	Type	Picture	Illustrate
GB1	WPC	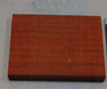	Recycled GBM thickness:25 mm Sawdust, PE
GB2	PS floor	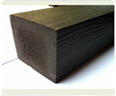	Healthy GBM thickness:90 mm PS, Styrene Acrylonitrile resin
GB3	WPC	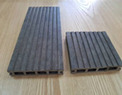	Recycled GBM Thickness:26.8 mm PP, PE + Wood
S1	PVC floor	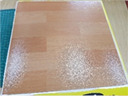	Non-GRM PVC
S2	SPC	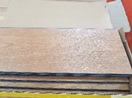	Non-GRM PVC
S3	PC	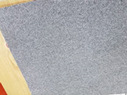	Non-GRM thickness:2.2 mm composite floor covered with carpet

**Table 5:** Results of phthalate concentration (μg/m^3^) in flooring building materials.

		DMP	DEP	DIBP	DBP	BBP	DEHP	DNOP	DINP	DIDP
GB1	STAGE 1	N.D.	N.D.	N.D.	N.D.	N.D.	N.D.	N.D.	N.D.	N.D.
	STAGE 2	N.D.	N.D.	18.288	50.685	27.816	630.802	40.766	43.643	N.D.
	SUM	N.D.	N.D.	18.288	50.685	27.816	630.802	40.766	43.643	N.D.
	Percentage of content			2.3%	6.2%	3.4%	77.7%	5.0%	5.4%	
GB2	STAGE 1	2.197	2.591	3.007	3.426	3.726	3.451	4.268	N.D.	N.D.
	STAGE 2	15.684	21.088	23.655	34.98	33.551	554.288	52.8	96.247	78.281
	SUM	17.881	23.679	26.662	38.406	37.277	557.739	57.068	96.247	78.281
	Percentage of content	1.9%	2.5%	2.9%	4.1%	4.0%	59.8%	6.1%	10.3%	8.4%
GB3	STAGE 1	N.D.	N.D.	N.D.	N.D.	3.178	N.D.	3.245	N.D.	N.D.
	STAGE 2	25.406	N.D.	N.D.	N.D.	20.534	1388.18	20.424	N.D.	N.D.
	SUM	25.406	N.D.	N.D.	N.D.	23.712	1388.18	23.669	N.D.	N.D.
	Percentage of content	1.7%				1.6%	95.0%	1.6%		
S1	STAGE 1	5.242	15.624	5.22	20.82	N.D.	N.D.	2.289	N.D.	N.D.
	STAGE 2	N.D.	N.D.	N.D.	14.444	N.D.	355.76	27.872	75.696	N.D.
	SUM	5.242	15.624	5.22	35.264	N.D.	355.76	30.161	75.696	N.D.
	Percentage of content	1.0%	3.0%	1.0%	6.7%		68.0%	5.8%	14.5%	
S2	STAGE 1	N.D.	N.D.	N.D.	N.D.	4.095	2.533	3.282	N.D.	N.D.
	STAGE 2	N.D.	N.D.	N.D.	N.D.	24.42	32.084	35.78	N.D.	N.D.
	SUM	N.D.	N.D.	N.D.	N.D.	28.515	34.617	39.062	N.D.	N.D.
	Percentage of content					27.9%	33.9%	38.2%		
S3	STAGE 1	N.D.	N.D.	N.D.	N.D.	4.856	4.51	3.397	72.612	N.D.
	STAGE 2	N.D.	N.D.	N.D.	23.021	26.501	438.238	20.176	N.D.	N.D.
	SUM	N.D.	N.D.	N.D.	23.021	31.357	442.748	23.573	72.612	N.D.
	Percentage of content				3.9%	5.3%	74.6%	4.0%	12.2%	

**Table 6:** Literature study and test results of phthalate in building materials.

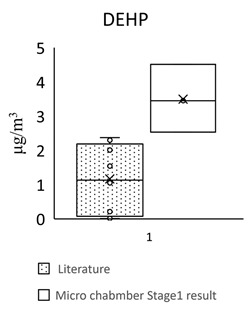	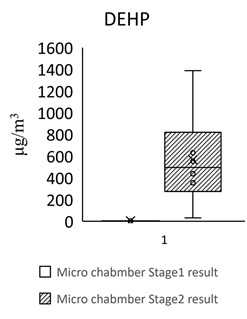	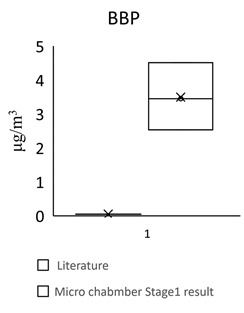
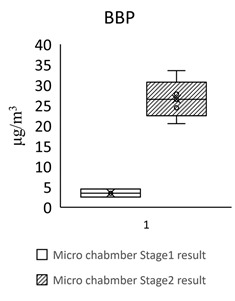	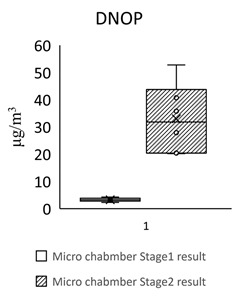	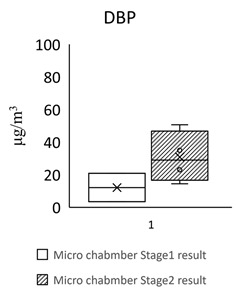

Literature study reference from Shanshan Shi et al. 2018 [42].
